# Evaluating the effects of lymphoedema management strategies on functional status and health-related quality of life following treatment for head and neck cancer: a systematic review

**DOI:** 10.1007/s11764-023-01453-7

**Published:** 2023-08-30

**Authors:** Lauren J. Mullan, Nicole E. Blackburn, Jackie Gracey, Lynn Dunwoody, Jill Lorimer, Cherith J. Semple

**Affiliations:** 1 School of Nursing, Institute of Nursing and Health Research, Ulster University, Belfast, UK; 2 School of Health Sciences, Institute of Nursing and Health Research, Ulster University, Londonderry, UK; 3 School of Psychology, Faculty of Life and Health Sciences, Ulster University, Londonderry, UK; 4 Physiotherapy Department, Cancer Centre, Belfast Health and Social Care Trust, Belfast, UK; 5 Cancer Services, South Eastern Health and Social Care Trust, Belfast, UK

**Keywords:** Head and neck lymphoedema, Head and neck cancer, Cancer survivorship, Adherence, Self-management, Systematic review

## Abstract

**Purpose:**

Patients living with head and neck lymphoedema (HNL) after completion of head and neck cancer (HNC) often can experience long-term functional challenges and overall poorer health-related quality of life (HRQOL). This systematic review aims to explore components of effective HNL interventions through identification and synthesising literature on existing HNL management interventions.

**Methods:**

Five electronic databases (MEDLINE via Ovid and PubMed, CINAHL, CENTRAL, and Scopus) were systematically searched using Medical Subject Headings and free text, as well as citation tracking and Google Scholar for grey literature.

**Results:**

A total of 1910 studies were screened, with 12 studies meeting the inclusion criteria. Findings indicated vast heterogeneity within HNL interventions. Patients’ adherence to intervention strategies was reported as low and partially adhered to, particularly at home. This impacted on function domains and overall HRQOL during the post-treatment HNC phase, as well as further increasing the demands placed on healthcare professionals.

**Conclusions:**

Synthesis of the research findings highlighted a need to provide and educate patients with individualised HNL self-management intervention strategies. Promoting adherence was reported as being essential, with self-efficacy and behaviour change techniques being emphasised as a critical element to enhance motivation and therefore effective intervention delivery. Further work is important to address barriers to adherence and promote both motivation and behaviour change, to develop individualised self-management interventions for this cancer population.

**Implications for Cancer Survivors:**

The findings from this systematic review will provide guidance in the development and delivery of individualised self-management HNL interventions for patients who have completed HNC treatment.

## Introduction

Head and neck cancer (HNC) is the 6th most common cancer globally, with more than 930,000 new cases diagnosed annually [1, 2]. Significant developments are continuing to be made in detecting, diagnosing, and treating HNC, with ever increasing numbers of individuals living longer with the associated cancer and treatment-related consequences [3, 4].

The treatments for HNC consist of multi-modality approaches using combinations of surgery, radiotherapy, and chemotherapy, along with the promising emergent role of immunotherapy [5, 6]. Despite these treatments being associated with significant improvements in survival rates, the acute and chronic side effects of HNC treatment are evidenced as having potentially detrimental effects on HNC patients’ post-treatment health-related quality of life (HRQOL) [7]. Head and neck lymphoedema (HNL) is increasingly identified as a common chronic side effect of HNC treatment, impacting both functional status and HRQOL domains [6, 8].

Despite HNL being a common consequence of HNC treatment, it is often under-recognised and under-treated [7]. This is a critical issue to address, as the prevalence of this condition can be as high as 90% [6]. HNL is a life-altering condition, which presents as an abnormal level of swelling and accumulation of protein-rich fluid in the interstitial spaces of the head, face, and neck, with research indicating that many HNC survivors experience the life-limiting effects of HNL on their daily lives with function domains and HRQOL being significantly affected [9, 10]. This may involve functional impairments such as swallowing challenges, eating difficulties, and restricted range of motion, alongside HRQOL impacts such as pain, loneliness, reduced quality of sleep, reduced social engagement, and body image issues [11].

Historically, there has been a lack of high-quality, evidence-based research to direct the prevention and management of HNL, as most intervention studies have been based on limb lymphoedema. Despite a recent expansion of studies within the HNC tumour group, there is still a dearth of direction for HNL management [12, 13]. Moreover, a recent review focusing on HNL assessment measures indicated that care for HNL is varied globally and there is no clinical pathway, single modality treatment, or process of referral [8]. Despite complete decongestive therapy (CDT) being deemed the ‘gold standard’ for the management of HNL, alternative treatment modalities have shown positive outcomes, to include liposuction, pneumatic compression, and Kinesio taping [6, 14]. Furthermore, the results from these HNL interventions are variable in terms of improvements in function and HRQOL, demonstrating lack of consensus and poor patient compliance within HNL management. Self-management has tentatively been stated to have a positive effect, after sufficient training, on HRQOL benefits on HNL [15]. Despite this, there is a lack of evidence-based intervention studies demonstrating its overall effectiveness [16]. Both self-management and compression therapy, which are key elements of HNL management, are hampered by issues surrounding poor adherence [6].

With an evident gap in the current body of literature surrounding effective HNL management strategies, there is a need to systematically collate and analytically synthesise the knowledge base on HNL intervention studies to ascertain efficacy for this population. This systematic review will identify, evaluate, and synthesise HNL intervention studies in order to draw conclusions surrounding effective HNL management strategies. Furthermore, these conclusions will potentially aid the identification of key areas to inform the planning, development, and delivery of evidence-based interventions, therefore addressing existing challenges surrounding HNL management.

More specifically, the aim of this systematic review is to investigate the effectiveness of HNL management strategies on functional domains such as speech, eating, trismus, and range of motion in the neck, shoulder, and jaw, and overall HRQOL following treatment for HNC patients. The objectives are to.

1. evaluate how effective HNL interventions are in relation to improving functional related outcomes for HNC patients.
2. evaluate how effective HNL interventions are in relation to improving HRQOL outcomes for HNC patients.
3. identify which components of HNL interventions are most effective for HNC patients.
4. identify aspects of HNL interventions that facilitate or inhibit engagement with or effective use by HNC patients.

## Materials and methods

### Data sources and search strategy

The systematic review adhered to a priori protocol according to the Preferred Reporting Items for Systematic Reviews and Meta-analyses (PRISMA) 2015 guidelines [17]. The review was registered on the International Prospective Register of Systematic Reviews (PROSPERO), registration number CRD42022378417.

A comprehensive and systematic search of the literature was conducted to identify studies relating specifically to HNL interventions. The key search terms were defined as ‘lymphoedema’, to include edema, odema, and swelling, and ‘head and neck’, to include head and neck cancer, head and neck neoplasms, and head and neck malignancy. The search terms used in this review were generated in collaboration with an experienced subject librarian and author (LM). Five databases were searched to identify the relevant literature; these were MEDLINE via Ovid and PubMed, Cumulative Index of Nursing and Health (CINAHL), Cochrane Central Register of Controlled Trials (CENTRAL), and Scopus. The review search strategy used both Medical Subject Headings (MeSH) terms and text word searches to enhance the sensitivity of the search. Boolean operators of ‘OR’ and ‘AND’ were chosen to combine the search terms to enable them to be broadened or limited as appropriate. The complete search strategy for Ovid MEDLINE has been included in Table 1. The results of the completed searches were collated in the software Covidence [18]. Grey literature was also searched through the medium of Google Scholar and citation searching of included study reference lists, to ensure any research studies not included in the electronic databases were identified.

**Table 1:** Search terms

Database: Ovid MEDLINE(R) ALL < 1946 to July 03, 2023 >
Search strategy:
1 Lymphedema/
2 (lymphedema or lymphoedema or edema or swelling).mp. [mp = title, book title, abstract, original title, name of substance word, subject heading word, floating sub-heading word, keyword heading word, organism supplementary concept word, protocol supplementary concept word, rare disease supplementary concept word, unique identifier, synonyms, population supplementary concept word, anatomy supplementary concept word]
3 1 or 2
4 ‘Head and Neck Neoplasms’/
5 (‘head and neck cancer*’ or ‘head and neck malignancy’ or ‘head and neck neoplasm*’ or laryngeal cancer or oral cancer or head cancer or neck cancer).mp. [mp = title, book title, abstract, original title, name of substance word, subject heading word, floating sub-heading word, keyword heading word, organism supplementary concept word, protocol supplementary concept word, rare disease supplementary concept word, unique identifier, synonyms, population supplementary concept word, anatomy supplementary concept word]
6 4 or 5
7 3 and 6
8 limit 7 to (english language and yr = ‘2002 –Current’ and ‘all adult (19 plus years)’)

Inclusion and exclusion criteria were carefully constructed and applied in the search strategy for this review. The initial search was required to be broad and inclusive to capture the inclusion of related studies. The inclusion criteria consisted of (1) participants being aged 18 and over; (2) primary studies including randomised control trials, feasibility, and pilot studies; (3) HNL management intervention studies; (4) English language; and (5) limits set between 2002 and 2022. This search limit was extended to 20 years to ensure no studies were excluded due to the initial scoping of literature demonstrating limited literature surrounding HNL management. An updated search was conducted in July 2023 to ensure no additional papers had been published since, but no other manuscripts were identified. This updated search strategy is evidenced in Table 1. Studies were excluded if they reported on more than one type of cancer, but HNC data could not be segregated and if articles were of secondary research such as reviews, editorials, and those utilising secondary data.

### Screening

**Fig. 1:**
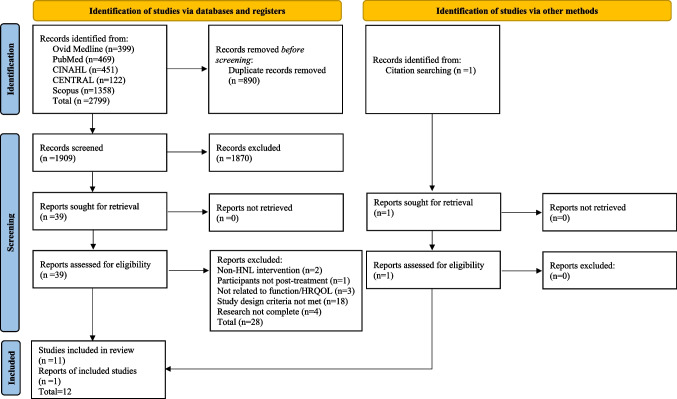
PRISMA flow chart

The initial search strategies identified 2799 studies which were imported to Covidence for screening, with 890 duplicates removed and one study included through citation searching. In total, there remained 1909 studies available to be screened. The title and abstracts were screened by the first author (LM) and independently screened by a second author (CS). As a result, there were 39 full texts remaining that met the eligibility criteria and one additional study through citation searching. Full-text papers were also independently screened using the eligibility criteria through Covidence by the same two authors, to ensure rigour. Two disagreements between undetermined studies were addressed through initial discussion between LM and CS and a decision was made, without the need for abirritation from an available third author (NB). In total, there were 12 studies included for complete data extraction. This screening process is outlined in a PRISMA flow chart in Fig. 1.

### Data extraction

The first author (LM) produced the data extraction form in Microsoft Word, with input from three other authors (CS, NB, LD). Data was extracted on the author(s), year, country, title of paper, study aim, research design, sample characteristics, intervention features, outcome measures, main findings, and barriers/facilitators of intervention. This can be viewed in Table 2. The data was independently extracted from the 12 included studies by LM and additionally reviewed by four authors (CS, NB, LD, JL) with expertise in evidence synthesis and or lymphoedema management. Any discrepancy in opinion surrounding the extraction of data was resolved through discussion with CS and NB. The first author (LM) independently carried out the quality appraisal of the evidence for each included study using the grading of recommendations, assessment, development, and evaluation (GRADE) tool and GRADEpro software. To enhance rigour, a second author (CS) independently completed GRADE for 10% of the included studies, with concordance gained. This GRADE assessment included five domains including risk of bias, inconsistency of results, indirectness, imprecision, and publication bias. The levels for certainty of evidence are rated as high, moderate, low, and very low.

**Table 2:** Summary of study characteristics

Author (s)	Study aim(s)	Study design	Sample	Intervention features	Outcome measures	Summary of findings	Challenges/barriers and/or facilitators
Ridner et al. (2020), USA [14]	Evaluate the feasibility and efficacy of using the Flexitouch system (FT), advanced pneumatic compression device (APCD) in HNC survivors who have HNL	Randomised waitlist controlled trial	*N* = 43 participants. Age range 56–68	FT intervention (INT) group and waitlist control group. INT group was self-management plus FT device twice daily for 8 weeks. Time for use based on size of garment and ranged from 23 to 45 min Waitlist control—usual care with option to continue with FT post study for 8 weeks	Head and neck lymphoedema and fibrosis assessment criteria, endoscopic exams, and modified Patterson scale, LSIDS-HN, the Vanderbilt head and neck symptom survey, 5-item linear analogue self-assessment, NDI, voice handicap index, cervical and shoulder ROM instruments, TheraBite jaw ROM scale, and CTCAE v4.0. All assessed at baseline and end of study at 8 weeks	(1) Trial supported safety and feasibility of the APCD in treating HNL. (2) Findings relating to adherence supported daily vs twice daily dosage. (3) FT significantly enhanced patients’ perception of their ability to control their HNL. (4) FT demonstrated positive but not significant effects on oral symptoms (swallowing and difficulty moving tongue) and activity (bending)	(1) Time factors were the most common barrier to non-adherence. Included family reasons (*n* = 3), work issues (*n* = 3), and travel (*n* = 2). (2) Discomfort related included pressure/metal issues (*n* = 1), noise (*n* = 1), garment fit (*n* = 1), and feeling unwell (*n* = 1) Facilitator included patient’s ‘perceived ability to self-manage’ critical outcomes for HNL therapy
McLaughlin et al. (2020), Australia [19]	To determine how feasible a self-management lymphedema randomised control trial would be in testing the effectiveness of a head and neck-specific exercise protocol	Pilot randomised pre-test and post-test control trial	*N* = 9 participants. Aged > 18, mean age 62 years	Active control arm—standard HNL care (CDT) and 45-min appointment for 6 weeks INT—additional 15 head and neck specific exercises and 60-min appointment	Diary logs, ALOHA tape measurement, MDACC HNL rating scale, EORTC QLQ-H&N43. All assessed at baseline and end of study at 6 weeks	(1) INT had low adherence due to increased burden of additional exercise protocol. (2) Future trials may consider including the use of technology to provide real-time guidance. (3) No significance between group differences or trend for body image and overall HRQOL	(1) Adherence issues with compression. (2) Low adherence due to increased burden of exercise INT. (3) Level of contact was not frequent enough. (4) Incorrect self-management techniques lowered adherence. (5) One face-to-face session and written handouts insufficient training. (5) Weekly telephone calls facilitated psychosocial support and emotional benefit
Author (s)	Study aim(s)	Study design	Sample	Intervention features	Outcome measures	Summary of findings	Challenges/barriers and/or facilitators
Pigott et al. (2021), Australia [20]	To determine the feasibility of a randomised control trial comparing MLD and compression to manage HNL	Randomised control trial feasibility study	*N* = 5 participants. Median age 54. Age range 40–80 years	MLD group—treated × 2 per day by HCP and performed MLD × 1 per day at home for 6 weeks. Compression group—3 weekly telephone calls from HCP and 3 face-to-face support appointments for 6 weeks. Use at home 8 h overnight for 6 weeks. Self-management 6–12 weeks	Moisture metre compact, MDACC HNL rating scale, ALOHA tape measurement, fibreoptic endoscopic evaluation of swallowing, Patterson’s radiotherapy oedema rating scale, EQ-5D-5L, EQ VAS, EORTC QLQ-C30, EORTC H&N43, distress thermometer, and diary logs. All assessments at weeks 1, 6, and 12. External HNL also at week 13	(1) Clinical outcomes did not provide strong levels of support for single modality treatments for HNL with % tissue water increasing in all participants at 12 weeks. (2) Compression appointments were less adhered to compare with MLD with 78.6% adherence compared to 91.7%. (3) Adherence to home programme was ‘partially adhered’ to. (4) Future studies require adaptations to design of study to ensure recruitment is increased	(1) Barrier to intervention was recruitment due to COVID-19 restrictions. (2) Adherence to home programmes were self-reported as ‘partially adhered’ to. (3) Daily time commitments of MLD lowered adherence. (4) Reduction in referrals to lymphoedema clinic was a barrier to recruitment. (5) Study was costly and required high levels of staffing to organise appointments, assessments, and treatment
Tsai et al. (2022), Taiwan [21]	Compare effects of early interventions with rehabilitation exercise in contrast to MLD and rehabilitation (rehab) exercise in terms of pain, ROM, and lymphoedema in oral cavity patients	Randomised single blind design	*N* = 39 participants. Average age of 55.5 years	MLD + rehab group—30-min MLD and 30-min rehab exercise on workdays Rehab only group—30 min on workdays. Initiated 7–10 days post-surgery	VAS, ROM of neck and shoulder instruments, Siemens Acuson Antares PE ultrasound, and face distance for lymphoedema, Földi and Miller lymphoedema scales, and tape measurements	(1) MLD and rehab effective in improving ROM in neck. (2) Potential therapeutic role for use of early intervention with MLD in addition to rehabilitation exercise had greater benefit in controlling HNL and improving neck ROM. (3) VAS pain score significantly improved after intervention	(1) Longer-term follow-up periods required as difficult to assess effects of early interventions in preventing further morbidities. (2) The severity of neck dissections acted as a barrier due to 7–10 sessions not sufficient to recover impaired ROM
Author (s)	Study aim(s)	Study design	Sample	Intervention features	Outcome measures	Summary of findings	Barriers and/or facilitators
Atar et al. (2022), Turkey [22]	To investigate the effectiveness of Kinesio taping (KTT) for lymphedema following HNC therapy and the effects on patient compliance and QOL	Randomised, double blind, sham-controlled trial	*N* = 66 participants. Mean age of KTT group was 51.47 years, and sham KT (SKT) was 51.11 years	KTT—lymphatic correction technique. SKT—no lymphatic correction but single strip use. Both involved 4-week MLD with home CDT. Week 1 performed × 5 days for 45 min and weeks 2–4 performed × 2 days	MDACC rating scale, fibreoptic endoscopic image and Patterson oedema scale, VAS, EORTC-QLQ-C30, and QLQ-H&N35. Assessments at baseline, 4 weeks, and 8 weeks	(1) KTT provided better results in composite tape measurement of external HNL and significantly better QOL parameters (global health status, pain, swallowing, social functioning, loss of appetite, social eating, and mouth opening). (2) KTT in combination with MLD is both safe and well tolerated by HNL patients. (3) Some patients did experience mild to moderate itching, redness of skin and tension	(1) Although no patients developed any injuries due to KTT, a small wound did develop in one patient in the HNL area localised in submental region where KTT was not adhered to due to beard shaving. (2) Patients with tracheostomy may prove challenging and recommended careful taping to avoid risking airways and prevent contact of KTT with secretions. (3) Leaving HEP to the responsibility of patients or carers was reported as a limiting factor, but no explanation or findings were included as to why
Ozdemir et al. (2021), Turkey [12]	Effectiveness of CDP when administered by physiotherapist or home-based programme on external HNL, and 3D surface scanning and volume evaluations	Randomised controlled trial	*N* = 20 participants. Mean age was 64.9. Age ranges from age 46 to 84 years	CDP INT—MLD × 5 days/30 min for 4 weeks, 4–6-h compression, exercises of 10 reps × 5 days for 4 weeks. Home-based programme—self MLD × 1 daily and exercises of 10 reps daily for 4 weeks Control—medical controls without INT	MDACC HNL protocol and rating scale, 3D scanner, and software to assess and calculate volume of HNL region. Assessments at baseline and 4 weeks later	(1) Significant decrease in CDP and home-based (*p* ≤ 0.05) for point-to-point measurements. (2) 3D volume analysis showed significant decreases in CDP group. (3) External HNL and fibrosis at submental region significantly decrease in CDP and home-based groups. (4) Home-based programme benefits those who cannot receive CDP INT due to insufficient numbers of certified therapists	(1) Not possible to compare INT to non-invasive measures due to unavailability of HNL physiotherapists
Author (s)	Study aim(s)	Study design	Sample	Intervention features	Outcome measures	Summary of findings	Challenges/barriers and/or facilitators
Alamoudi et al. (2018), Canada [23]	To review the outcomes of head and neck cancer patients who had submental liposuction for their post-treatment lymphoedema and compare the results with a control group	Randomised controlled trial	*N* = 20 participants. Mean age was 64.9. Age range 46–84 years	INT—liposuction submental procedure Control—6-month waiting period without surgery	DAS-59 and MBOE. Surveys prior to liposuction procedure, again at time of surgery, and 6 months or more following the procedure	(1) Study demonstrates statistically significant improvements in overall QOL in DAS-59 scale with patients after receiving liposuction. (2) Liposuction showed statistically significant improvement in self-perception and self-confidence of appearance using the MBOE and DAS-59 scales	(1) Barrier was that it was not possible to compare INT to non-invasive measures such as physiotherapy due to the unavailability of dedicated physiotherapists dealing with HNL. (2) Patient selection in submental liposuction can be limited by patient age, skin tone, position of hyoid, or amount of fat deposit on physical exam. However, in this study, patient selection did not have these limitations but rather all were included if they were medically fit, free of disease and interested in the procedure
Deng et al. (2022), USA [24]	To determine feasibility and preliminary efficacy data of a multifaceted self-care programme (SCP) for lymphoedema and fibrosis (LEF)	Pilot randomised clinical trial	*N* = 59 participants	Group 1—usual care with monthly telephone calls for 12 months Group 2—usual care plus LEF-SCP. Training > 1 h for 3 weeks. Duration 12 months? Group 3—usual care plus LEF-SCP and routine follow-up with HNL therapist > 1 h every 3, 6, and 9 months. Duration 12 months	LEF progression, symptom burden and functional status, PMCSMS, and self-care checklist	(1) LEF-SCP was feasible for HNC survivors with 80% completion rate and 90% satisfaction rate. (2) Preliminary efficacy demonstrated in LEF-SCP group with a significant decrease in LEF severity, symptom burden. (3) No significant improvement in jaw ROM with LEF-SCP	N/A
Author (s)	Study aim(s)	Study design	Sample	Intervention features	Outcome measures	Summary of findings	Challenges/barriers and/or facilitators
Yao et al. (2020), USA [25]	To compare the outcomes in patients with HNL who receive either a home-based programme or a hybrid approach including both home-based and regular clinic visits	Pilot study: pre- and post-treatment assessment	*N* = 50 participants. Home therapy group had mean age of 57.36. Hybrid group mean age was 66.76	Home training group—CDT protocol for approximately 99 days Hybrid group—daily treatment of CDT and weekly clinic visits of 30–40-min appointments. Duration approximately 99 days	MD Anderson HNL rating scales, tape measures, and composite scores. Assessments at baseline, prior to implementation, and approximately 3 months post-treatment	(1) There were comparable improvements in HNL measures in both groups (66% participants). (2) Home-based treatment was suggested to be appropriate if patients were unable to participate in clinical sessions. (3) Participants with facial oedema or more advanced stage HNL may benefit from clinician-led approaches. (4) Adherence was greater in hybrid treatment (84%) than home-based (68%)	(1) Limited availability of local therapists, transport issues, and lack of outpatient benefits. (2) Adherence was more favourable in patients receiving some level of clinician intervention
Nixon et al. (2018), Australia [26]	To examine the course and nature of distress and person-centred experience of HNL	Mixed method explanatory design	*N* = 10 participants. Mean age of 65.1. Age range was 42–89 years	Component 1—22-week therapist directed physical HNL programme and self-management with prospective repeated measures examining distress, physical ssocia and QOL Component 2—qualitative interviews 10–22 months post 22-week HNL programme	Distress thermometer and EORTC QLQ-H&N43. Assessments at baseline, week 6, and week 22 (end of study). Semi-structured interviews at 10–22 months after completion of the 22-week HNL programme	(1) Distress ssociate with cancer treatment-related HNL reduced significantly with delivery of a HNL programme. (2) All participants referred to HNL impacting their functional capacity. (3) HNL negatively impacted appearance. (4) MLD and compression appeared to be most beneficial with patients. (5) No significant differences in QOL, body image, or fear of recurrence	(1) Barrier to implementation necessitate qualitative study design. (2) Different methodologies, using telephone and face-to-face, can have the potential to impact differently on the content expressed by participants
Author (s)	Study aim(s)	Study design	Sample	Intervention features	Outcome measures	Summary of findings	Barriers and/or facilitators
Deng et al. (2021), USA [27]	To determine if the use of photo-biomodulation (PBM) therapy has feasibility and potential efficacy for HNL, symptom burden, and head and neck range of motion among HNC survivors	Pilot feasibility study: single arm pre- and post-design clinical trial	*N* = 12 participants. Mean age 58 years	INT—PBM therapy × 2 weekly for 6 weeks. *N* = 14–25 points on face and neck treated. Total of *n* = 12 treatments. Prior to PBM 5 min of MLD	HN-LEF symptom inventory, neck disability index, cervical range of motion device, and modified Patterson scale. Assessments at baseline, end of treatment, and 4 weeks post-intervention	(1) PBM therapy showed significant reduction in severity of external HNL. (2) Significant decrease with PBM in symptom burden of jaw/oral dysfunction, swallowing, body image, sexuality, and communication between baseline and end of INT. (3) A significant increase found with PBM in cervical ROM between baseline and 4 weeks post-INT. (4) Adherence was demonstrated as participant attrition was low (8.3%) with only *n* = 1 participant unable to complete the study due to prior family engagement	(1) Potential barrier was frequency of sessions, therefore concerns about intervention duration, feasibility, and adherence. (2) Participant burden was minimised, and participation was facilitated through design of intervention with it being convenient for participants and ensuring each session did not exceed 30 min. (3) A facilitator for intervention was decreased frequency of travelling in design of study as study visits (assessments and treatments) were scheduled at a convenient time for participants or on days that they were attending a routine oncology appointment
Author (s)	Study aim(s)	Study design	Sample	Intervention features	Outcome measures	Summary of findings	Challenges/barriers and/or facilitators
Bruns et al. (2004), Germany [28]	To evaluate the effects of selenium in the treatment of lymphedema of the head and neck region	Pre- and post-treatment exploratory design study	*N* = 36 participants. Median age 61 years	INT—350 µg/m^2^ body surface sodium selenite p.o. daily, generally resulting in total dose of 50 µg/m^2^ per day. Treatment over 4–6 weeks, with median of 5 weeks. No patient received additional medications	Late Effects Normal Tissue Task Force- Subjective, Objective, Management and Analytic (LENT-SOMA) scoring system, scoring system of Földi and Miller system, and VAS. Assessments at baseline and after selenium treatment. Maximal treatment effect recorded 4 weeks after end of treatment	(1) Positive effect of sodium selenite on secondary HNL caused by radiotherapy alone or combined after surgery. (2) Larger RCT required with addition of a control group and longer follow-up to verify findings. (3) Selenium appears to be cost-effective in contrast to HNL physical therapy	N/A

*LSIDS-HN* lymphoedema symptom intensity and distress survey-head and neck, *NDI* neck disability index, *ROM* range of motion, *CTCAE* Common Terminology Criteria for Adverse Events, *EORTC QLQ-H&N43* European Organisation for Research and Treatment of Cancer Quality of Life Questionnaire Head and Neck Module 43, *EQ-5D-5L* European Quality of life five dimension five level version, *EORTC QLQ-C30* European Organisation for the Research and Treatment of Cancer Quality of Life Module 30, *EORTC QLQ-H&N35* European Organisation for Research and Treatment of Cancer Quality of Life Questionnaire Head and Neck Module 35, *VAS* visual analogue scale

### Data synthesis

Due to heterogeneity of outcome measures across the included studies, variety of study designs, and interventions being tested within studies, a narrative synthesis was conducted to synthesise the findings of the 12 included studies. The narrative synthesis was conducted by developing a preliminary summary of the intervention studies from the data extraction table, and the main findings that were in numerical form were translated into text. Relationships within and between studies were explored in relation to study objectives. This included exploration of the variability of outcomes reported in the data according to study design, intervention characteristics, implementation, or delivery.

## Results

### Study characteristics

The 12 included studies contained heterogeneity within study design, including randomised control trials (*n* = 5), feasibility studies (*n* = 5), and mixed method studies (*n* = 2). Within these studies, there was a heterogeneous HNC population that involved a range of different tumour sites such as oropharynx, oral cavity, salivary glands, larynx, infraorbital, hypopharynx, nasal cavity, and thyroid. There was also a diverse range of treatment modalities received by participants and included cancers at different stages. Sample sizes ranged from between six [20] and sixty-six [22], with the majority of studies reporting a predominantly male population. Included studies were conducted across a range of different countries, including four in the USA [14, 24, 25, 27], three in Australia [19, 22, 23], two in Turkey [12, 25], and the remainder in Canada [23], Taiwan [21], and Germany [28]. There were a range of both clinician-rated outcomes and patient-reported outcome measures across the studies.

A heterogeneous range of tools were used to measure both external and internal lymphoedema. The MD Anderson Cancer Centre Head and Neck Lymphoedema (MDACC HNL) rating scale [15] and tape measurements were the most popular tools to measure external HNL with six studies using the MDACC HNL method. Other tools used to measure external HNL included the Assessment of Lymphedema of the Head and Neck (ALOHA) [19], Földi and Miller lymphoedema scales [29, 30], percentage water content moisture metre compact [20], 3D scanner for volume of HNL region [12], and touch/visual examination. Internal lymphoedema was measured in four studies using a fibreoptic endoscopic examination, with further two studies using the modified Patterson scale [31] and two others using the Patterson oedema scale [32]. Further heterogeneity was apparent, arising from the different treatment modalities for HNC within the included studies contributing to different impacts on participants’ functional status. These included voice changes, trismus, cervical, and jaw range of motion [14, 24, 27], as well as neck and shoulder range of motion [21, 27] and swallowing [14, 19, 22, 27].

Heterogeneity also existed in the tools used to measure HRQOL. Three studies incorporated the use of the EORTC QLQ-H&N 43 tool [19, 20, 26] and two studies used the EORTC QLQ-C30 [21, 22]. Additional HRQOL outcomes were assessed using the EORTC QLQ-H&N 35 [22], EQ-5D-5L [20], EQ visual analogue scale [20, 22], positive and negative affect scale (PANAS) [24], Derriford Appearance Scale (DAS-59), and Modified Blepharoplasty Outcome Evaluation (MBOE) for patient perceptions of appearance [23] and the distress thermometer [20, 26].

### Summary of interventions

Within the included studies, there was a wide range of interventions incorporated to manage HNL. Three studies included self-management in the treatment of HNL; however, they all used different components including exercise protocols [19], lymphoedema and fibrosis self-care regimes [24], and self-administered manual lymphatic drainage (MLD) and home exercises [12]. Two studies had similarities as the interventions had a focus on MLD. These two studies did demonstrate evident differences in that one compared MLD with compression [20] whereas the second study assessed the effects on MLD when used early but only within oral cavity cancers [21]. The remainder of studies involved a range of different interventions for the treatment of HNL which included advanced pneumatic compression [14], home-based HNL management programmes [25], submental liposuction [23], photo-biomodulation therapy [27], Kinesio taping [22], HNL clinician-led treatment programme measuring distress and person-centred experience [26], and selenium treatment [28].

It is important to note that all included studies recruited participants who had completed treatment for HNC and were therefore in the post-treatment phase of their cancer journeys. Despite this similarity, the time from completion of treatment varied ranging from 0–6 to 12 months. The frequency and mode of intervention delivery demonstrated vast heterogeneity. Across the 12 studies, the duration of intervention study ranged from four weeks [12] to 12 months [24].

### Grading of recommendations, assessment, development, and evaluation

The overall quality of evidence for the randomised control studies was rated as very low which suggested a low level of confidence in the effect estimate. As a meta-analysis was deemed inappropriate, effect sizes or details on how wide confidence levels were have not been reported. Using GRADE, serious concerns were demonstrated with regard to the levels of heterogeneity and risk of bias. This can be viewed in the summary of findings (Table 3). Within risk of bias, most studies did report details on random generated sequence allocations; however, there was an overall high risk of bias due to concealments not being made clear, lack of blinding associated with outcome measures, and lack of detail surrounding participant allocation. It is of note that due to the nature of these intervention studies, it can be challenging to achieve blinding of participants and outcome assessors because of most outcomes being patient reported. The table of the summary of findings clearly depicts the vast heterogeneity between interventions delivered in the studies and outcome measures used, as well as methodological heterogeneity in study design. Variability was found in the direction of outcomes in relation to a positive or negative effect.

**Table 3:** Summary of findings

Summary of findings			
Head and neck lymphoedema management strategies compared to control for head and neck cancer patients			
Patient or population: HNL patients who have completed treatment for HNC Intervention: HNL management strategy Comparison: control			
Outcomes	Number of participants (studies)	Impact	Certainty of evidence (GRADE)
Functional domains	312 (6 randomised control studies)	In three studies, improvements in swallowing function were reported with intervention when compared to control groups. Only one study reported no significant difference in swallowing function with an exercise intervention. This was a small sample study and therefore may have contributed to the result. Range of motion (ROM) was reported in five studies. Only two studies reported statistically significant improvements in ROM of the neck and shoulder. Three studies demonstrated that although there were improvements shown with interventions in jaw, shoulder, and neck ROM, these results were not statistically significant when compared to control groups providing usual care. Interventions that significantly improved ROM included photo-biomodulation therapy and manual lymphatic drainage combined with rehabilitation	⨁◯◯◯ Very low^a,b,c^
Health-related quality of life (HRQOL)	278 (7 randomised control studies)	Four studies reported significant improvements in HRQOL including pain, social functioning, body image/appearance, and emotional functioning. Emotional and social functioning were improved with the use of Kinesio taping and photo-biomodulation. Acupuncture was recommended for consideration as significant improvements were found in self-perception and self-confidence. Two studies described statistically significant improvements in pain scores using MLD and Kinesio taping	⨁◯◯◯ Very low^b,c,d^
Adherence	173 (5 randomised control studies)	Majority of studies found low adherence. One study recorded an overall lack of compliance with a twice daily intervention regime and recommended a future once daily regime to encourage adherence. Adherence also reported in one study as ‘partially adhered’ to with tasks being performed less frequently than intended. Other studies demonstrated low adherence with compression devices and in an intervention associated with additional burden of exercise. Two studies reported benefits of adherence being associated with clinical improvements. At least half of participants in home-based programmes had 68% experiencing improvement and 84% in the hybrid intervention group. There were 72% adherent participants experiencing clinical improvements compared to only 28% in those not adhering to the intervention regimes in one study	⨁◯◯◯ Very low^c,d,e^

**GRADE working group grades of evidence**

*High certainty***—**we are very confident that the true effect lies close to that of the estimate of effect

*Moderate certainty***—**we are moderately confident in the effect estimate: the true effect is likely to be close to the estimate of effect, but there is a possibility that it is substantially different

*Low certainty***—**our confidence in the effect estimate is limited: the true effect may be substantially different from the estimate of effect

*Very low certainty***—**we have very little confidence in the effect estimate: the true effect is likely to substantially different from the estimate of effect

**Explanations**

^a^Most studies reported details on random generated sequence allocations; however, concealments were not made clear in most studies. Only two studies reported concealment details. Blinding was only reported in two studies, including blinding of assessors and participants in recruitment stage. Two studies did not provide any details on allocation of participants. Most studies dealt with missing outcome data adequately

^b^There was vast clinical heterogeneity across the intervention studies and in outcomes. Across most studies, there were differences in study design and methods. Within the reported functional domain outcomes, there was variability in the direction of the outcome in relation to positive or negative effect

^c^Narrative synthesis was conducted; therefore, estimates were not precise. There are no effect sizes or details on how wide confidence intervals are as this was a narrative synthesis

^d^Most of the studies reported on the random sequence generation, but concealments were not clear in over half of the included studies. Due to the nature of the included studies, blinding of participants and study personal was not possible after the allocation process. Overall, most studies avoided inclusion of describing the blinding process of assessors. Most studies dealt with missing outcome data sufficiently

^e^Vast heterogeneity in adherence with study design and direction of outcome effect

### Effect of HNL interventions in improving function-related outcomes for HNC patients

More than half of the studies included in this review specifically referred to function-related outcomes in exploring their effectiveness of interventions on HNL post-treatment. These functional outcomes included swallowing [14, 19, 22, 27], oral dysfunction [14, 27], speech difficulties [20], and range of motion of the neck, shoulder, and jaw [14, 19, 21, 22, 24, 27]. Statistically significant improvements in swallowing were reported by three different intervention studies from baseline to end of intervention [14, 22, 27]. These interventions involved advanced pneumatic compression treatment devices, photo-biomodulation treatment, and Kinesio taping. Deng et al. [27] also highlighted a statistically significant improvement in oral dysfunction; however, Ridner et al. [14] reported that although improvements were not statistically significant, patients did report improvements in their ability to move their tongue. McLaughlin et al. [19] conducted an intervention using a self-management exercise protocol but reported no statistically significant differences between usual treatment and intervention group for swallowing. It is of note that in this particular study, five out of nine participants did report improvements in their swallow function; however, two were from the intervention group and three from the usual treatment control group; therefore, there were no significant group differences.

Improvements in range of motion were reported in three of the included studies [21, 24, 27]. Both Deng et al. [27] and Tsai et al. [21] demonstrated statistically significant improvements within intervention groups in improving range of motion of the neck and shoulder and degree of cervical range of motion. Deng et al. [27] stated that their intervention of using photo-biomodulation therapy showed statistically significant results in the degree of participants’ cervical range of motion between baseline and 4-week post-intervention. The study completed by Tsai et al. [21] found that incorporating early intervention of MLD and rehabilitation exercises resulted in statistically significant improvements in neck and right shoulder range of motion. It is of note that improvements using MLD were superior to that of the rehabilitation exercise group. Although not significant, Deng et al. [24] did suggest improvements in jaw range of motion when using a lymphoedema and fibrosis self-care intervention.

### Effect of HNL interventions in improving health-related quality of life outcomes

The majority of included studies reported on HRQOL, but a varied range of outcome domains were assessed. These included distress [20, 23, 26], appearance/body image [19, 23, 26, 27], sexuality [23, 27], pain [14, 21, 22], global health [22], emotion [22, 27], social [22, 23], insomnia [22], and eating [22].

A synergistic finding in two studies was a significant improvement in body image/appearance [23, 27], from baseline to end of intervention, with liposuction and photo-biomodulation, respectively. Moreover, liposuction [23] indicated significant improvements in general self-consciousness, social self-consciousness, negative self-concepts, sexual and bodily self-consciousness of appearance, and facial self-consciousness of appearance. In contrast, two studies [19, 26] described no consistent pattern of improvement in body image within their interventions, HNL treatment intervention of therapist-directed treatment using massage, compression and exercise, and an exercise self-management intervention, respectively. It was reported within the exercise self-management intervention that this posed as an extra burden on participants, potentially contributing to the lack of symptom improvement and motivation and participants demonstrating incorrect self-management techniques of MLD and exercise at follow-up appointments [19].

Pain was reported to significantly improve in an intervention using MLD and rehabilitation techniques including coughing, breathing, and ROM of the neck and shoulder exercises [21] and with Kinesio tape [22]. One other study [14] also reported improvements in pain; however, these were not statistically significant. This study included an intervention using advanced pneumatic compression devices, which was associated with the stabilisation of pain in contrast to participants in the control group experiencing worsening in their pain over the course of the study from baseline to end of intervention. Sexuality concern was a less frequently reported HRQOL domain but was included by Alamoudi et al. [23] who showed significant improvements after the use of a liposuction intervention and Deng et al. [27] who also demonstrated significant improvements with photo-biomodulation.

Kinesio taping was shown to be effective in improving a number of different HRQOL domains, such as global health, emotional function, social function, fatigue, insomnia, and appetite loss using the EORTC-QLQ-C30 [22]. The same study also incorporated the EORTC-QLQ-H&N35 questionnaire which evidenced significant improvements in pain and eating.

### Adherence of HNL interventions

Adherence was reported in five out of the 12 included studies [14, 19, 20, 24, 25], of these three stated it to be low [14, 19, 20]. Ridner et al. [14] commented that participants used advanced pneumatic compression devices less than the prescribed twice a day intervention regime, with most only using it once. Pigott et al. [20] emphasised that adherence to home programmes was only partially adhered to and that adherence was greater with MLD than use of compression. With regard to MLD and compression, 26% of participants were found to adhere fully to MLD in contrast to 2.5% fully adhering to using compression [20]. In relation to exercise self-management, low adherence for most participants was thought to be due to the potentially increased burden placed on the intervention group [19]. These two studies clearly demonstrated synergy in their findings of compression having poorer adherence when compared to other HNL intervention management strategies [19, 20].

In contrast, two studies [24, 25] reported satisfactory adherence to interventions of lymphoedema and fibrosis (LEF) self-care programmes and home-based HNL programmes, respectively. When using a LEF self-care programme, there was good adherence with 80% completion rate and 90% satisfaction with both intervention groups and furthermore no difference between the intervention groups who experienced a follow-up appointment with a lymphoedema specialist and those who did not. In the home-based programme, the authors stated that adherence to treatment was linked with clinical improvements [25]. The author emphasised that at least half of the participants reported adherence in 68% of home-based treatments and in 84% of hybrid treatments, which involved a mixture of both clinician-led and home-based treatments [25]. This demonstrated that hybrid treatments are associated with greater rates of adherence [25].

### Facilitators and barriers to interventions

Despite the heterogeneity that existed within the included studies, there was synergy in identified barriers and facilitators to the interventions. Time-related factor was a common barrier. Ridner et al. [14] highlighted that time was the most common barrier associated with non-adherence in the intervention group using advanced pneumatic compression devices, including family reasons, work, and travel. In this intervention study, participants were reported to not adhere to the twice daily prescribed regime but rather only used the advanced pneumatic compression once a day. Interestingly, adherence to compression was also a common barrier to intervention implementation [12, 14, 19], with poor adherence relating to participants finding it complex to perform and experiencing discomfort [12, 14]. Additional barriers to intervention implementation involved frequency of contact with professionals [19, 27].

McLaughlin et al. [19] reported on adherence of their self-management exercise regime, suggesting that a single face-to-face session with written handouts was not sufficient to teach self-management techniques, potentially impacting overall motivation of participants to adhere and acting as a barrier to effective intervention delivery as patients were returning to follow-up appointments demonstrating incorrect techniques of MLD and exercises. Deng et al. [27] offer support in their study involving photo-biomodulation, by highlighting that the frequency of sessions used raised concerns with participants, in that too many sessions placed an added burden on HNL patients and negatively impacted their adherence, as well as the feasibility of the intervention.

As well as barriers to intervention implementation, the included studied also reported facilitators. One facilitator was emphasised as a participant’s perceived ability to self-manage vital outcomes for lymphoedema therapy [14]. By empowering participants, it has been suggested that this can encourage adherence to self-management and consequently lower long-term costs through the reduction of requirement for professional therapy regimes [14]. Furthermore, when participants received weekly telephone calls during a self-management intervention, this provided not only an emotional benefit to participants but also psychosocial support [19].

Minimising subject burden was raised as an important factor in facilitating interventions in the included studies. Deng et al. [27] described that this was possible through thoughtful patient-focused design of the intervention with appointments, sessions, and assessments being convenient to those participating and ensuring sessions did not last longer than 30 min in length. Convenience for participants was key as a facilitator in the photo-biomodulation therapy intervention by Deng et al. [27] by reducing travel commitments as visits for appointments and treatments were organised on days that participants already had routine oncology appointments and therefore did not interfere with daily life. One study reported that within a home programme intervention, group videos of self-management using MLD and exercises and written handouts were shared with both patients and their relatives to promote accurate and regular intervention adherence [12]. It was of note that despite this, there was no reference made to the impact of this on intervention delivery and adherence within the study’s findings [12].

## Discussion

To our knowledge, this is the first systematic review to synthesise the effectiveness of HNL management strategies for patients who have completed treatment for HNC. The timeliness of this landmark systematic review is important, evidenced by 11 of the 12 included studies conducted in the past 5 years, demonstrating an emergent area of research. This review clearly demonstrates that a vast degree of heterogeneity exists across HNL intervention studies in design, intervention type, duration, and outcome measures used, therefore making it challenging to define what the components are of an effective HNL intervention. Furthermore, the small number of available HNL intervention studies denotes the infancy of this field of inquiry. This is supported by Starmer et al. [8] who highlighted that the standard of care for HNL globally is varied, with no standard clinical pathway or process of referral clearly outlined for healthcare professionals and patients, demonstrating the need for improved evidence-based HNL management for this population [8]. Nonetheless, this review highlights some promise on the effectiveness of HNL interventions to improve HRQOL and functional outcomes, which is paramount given the high propensity (up to 90%) of HNC survivors living with this late effect of treatment [33]. Importantly, a few HNL interventions with self-management components pointed towards some benefits, which is encouraging given the requirement for long-term management of HNL [12, 19, 24], but adherence often proves challenging [12, 19]. Findings from this review depict the need for researchers to explore how adherence to HNL interventions can be improved, alongside an essential need for well-designed and reported studies, informed by theory, and guided by a complex intervention development framework [34, 35] and evaluated using validated tools such as EORTC-QLQ-43 to compare effectiveness of treatment components.

As noted above, the findings of this review highlight that despite some interventions demonstrating improvements in specific functional aspects of HNL, nonetheless there was no consistent trend evident. This is critical, as functional challenges to speech, eating, and range of motion frequently confronted by patients living with HNL result in them being placed at a greater risk of social isolation and poor overall HRQOL [36]. Although overall HRQOL was reported in most included studies within this review, there was heterogeneity within specific domains depicting improvements and no consistent trend in responses to specific interventions. At baseline body image/appearance, pain, swallowing, and range of motion were all commonly reported side effects of HNL in this review, emphasising the significant and detrimental impact this chronic condition can have on not only function but overall HRQOL if left untreated [23, 27].

Improvements within healthcare provision, to include treatment of HNC, have resulted in a greater number of people living longer with HNC as a chronic illness, requiring adaptations and changes to individuals’ lifestyles [37, 38]. Approaches to managing chronic illnesses including HNL have shifted from traditional provider/patient relationships to individuals playing a key role in their care through self-management [37]. Despite support from literature regarding the importance of promoting self-management to individual’s responses to HNC treatment-related side effects, [12, 19, 24], and not incorporating a ‘one size fits all’ approach [39], there is a lack of evidence within this review on how best to embed HNL self-management intervention strategies [39]. This is possibly due to the lack of clarity surrounding conceptualising of self-management within cancer care [40]. Nonetheless, recently, Dunne et al. [4] noted that there was evidence to suggest that self-management may be optimised within the context of an individualised approach. This individualised approach is supported by other researchers, who comment that an individual response to cancer-related treatment effects is necessary to aid adjustments to daily life following treatment for HNC [41, 42]. Given the chronicity of HNL [41] and the uniqueness of individuals and their cancer journeys, it may prove useful to provide HNL interventions promoting self-management strategies tailored to individual’s needs.

In the present review, adherence is highlighted as an important factor in effective HNL intervention delivery. Despite adherence being linked with clinical improvements, indication in this systematic review was that most HNL interventions were poorly adhered to [14, 25, 27]. There was evidence to suggest that issues surrounding adherence were associated with participants experiencing difficulties with compression devices, reporting they were too complex and caused discomfort [14, 20]. This association between poor adherence and compression is resultant from the unique head and neck anatomy and discomfort experienced by HNC patients [4]. Other factors contributing to poor adherence within this current systematic review are patient burden due to time-related barriers and frequency of contact for dose delivery of the intervention [12, 14]. Understanding and promoting adherence to HNL interventions warrants further exploration as an enriched understanding could, in part, mitigate the detrimental impact that HNL can have on patients’ function and overall HRQOL [43, 44].

In the current review, the effects of appearance were reported to be potentially detrimental and a commonly reported side effect [23, 27]. This is vital to consider within effective intervention delivery as changes to a person’s body image, because of HNL, can be difficult to hide and often create body image–related distress, therefore having the potential to present psychosocial issues, inhibiting the ability to self-manage their chronic HNL [44, 45]. Interestingly, the importance of self-efficacy within effective intervention delivery was emphasised in the current review and is an important component to consider when developing a HNL intervention [46]. Self-efficacy has been described as a strong predictor of how effective an individual will be in performing a given task and has a direct impact on achieving their goals [47]. A facilitator to self-managing chronic diseases such as HNL is to increase an individual’s self-efficacy to ensure they believe that they can manage their disease and therefore provide motivation and behaviour change [14, 48]. An individual’s self-efficacy is clearly identified as a facilitator in effective intervention delivery and may have the potential to have a positive impact on the low adherence rates reported in the current review and reduced demand placed on professional therapists, which is often a finite resource [4, 14].

An element of integrating self-management into daily life and increasing self-efficacy has been described as including techniques of trial and error and goal setting [38, 49]. There is synergy within previous research focusing on the inclusion of goal setting within behaviour change techniques. The concept of behaviour goal setting has been highlighted as key within behaviour change interventions [50]. This concept when implemented as a detailed plan, including the when, where, and how aspects of performing a behaviour proved to be more effective, results in positive changes within self-efficacy and behaviours in interventions [51, 52]. Patients with HNL may benefit from the integration of individualised behaviour goal setting, improving their adherence to specific self-management strategies and therefore promoting positive long-term behaviour change.

Considering the biopsychosocial challenges that accompany HNL, there is a dearth of information demonstrated within the current review on how individuals successfully can integrate self-management into their daily life. Relationships between patients and their family, friends, and healthcare providers is emphasised as being fundamental to successful self-management within chronic conditions [49]. In fact, those patients with greater levels of support from family members have demonstrated greater adherence to self-management techniques and control over chronic conditions such as HNL [39]. Despite this emphasis on the important role that family and healthcare providers can have, there is a paucity within this current review on how these roles can be utilised to deliver effective HNL interventions and promote adherence of self-management. Despite one study identifying the potential positive role that family members can have in promoting adherence and accurate self-management techniques, this potentially beneficial nature of family member support was not included as an outcome in the study [12]. Further research is required with patient and public involvement integration to develop effective HNL self-management interventions, which promote adherence and meet the biopsychosocial challenges presented by this patient population [12].

## Strengths and limitations

This systematic review was rigorously conducted with an extended publication criteria of 20 years to ensure no relevant studies were excluded. The inclusion and exclusion criteria resulted in studies that did not segregate HNC data from other cancer subtypes being excluded, therefore potentially being viewed as a limitation.

The initial searching of literature in this current review was performed by the first author (LM) and undertaken independently by a second author (CS) to ensure rigour, with data extraction performed by the first author only and checked by three co-authors (CS, NB, LD). Quality assessment of the included studies was conducted but no study being excluded due to poor quality due to the recent emergence of a small number of extant studies (*n* = 12). This could be viewed as a limitation as there were studies rated at a serious risk of bias with a very low level of certainty using the GRADEpro software tool.

## Conclusion

Effective intervention to manage the chronic effect of HNL for patients, although showing promise, is in its infancy. This current systematic review has demonstrated a dearth of literature focusing on the components of an effective HNL intervention, to include the role of self-management. Self-management has been reported to be beneficial in the long-term management of chronic conditions, including HNC, but adherence to home-based HNL interventions is generally poor. It is important to gain a richer understanding of what promotes long-term behaviour change to improve adherence to HNL interventions, to include the role of family members. Additionally, the impact and significance of this systematic review demonstrates a timely need to develop and evaluate evidence-based, theory-driven interventions with patient and public involvement embedded, to improve HNC patients’ functional outcomes and promote HRQOL as a consequence of HNL.

## Data Availability

The datasets generated during the current systematic review are available from the corresponding author on reasonable request.
